# Triple-negative breast cancers are increased in black women regardless of age or body mass index

**DOI:** 10.1186/bcr2242

**Published:** 2009-03-25

**Authors:** Lesley A Stead, Timothy L Lash, Jerome E Sobieraj, Dorcas D Chi, Jennifer L Westrup, Marjory Charlot, Rita A Blanchard, John C Lee, Thomas C King, Carol L Rosenberg

**Affiliations:** 1 Department of Medicine, Boston University Medical Center, 650 Albany Street, Boston, MA 02118, USA; 2 Department of Epidemiology, Boston University School of Public Health, 715 Albany Street, Talbot Building, Boston, MA 02118, USA; 3 Department of Pathology and Laboratory Medicine, Boston University Medical Center, 650 Albany Street, Boston, MA 02118, USA

## Abstract

**Introduction:**

We investigated clinical and pathologic features of breast cancers (BC) in an unselected series of patients diagnosed in a tertiary care hospital serving a diverse population. We focused on triple-negative (Tneg) tumours (oestrogen receptor (ER), progesterone receptor (PR) and HER2 negative), which are associated with poor prognosis.

**Methods:**

We identified female patients with invasive BC diagnosed between 1998 and 2006, with data available on tumor grade, stage, ER, PR and HER2 status, and patient age, body mass index (BMI) and self-identified racial/ethnic group. We determined associations between patient and tumour characteristics using contingency tables and multivariate logistic regression.

**Results:**

415 cases were identified. Patients were racially and ethnically diverse (born in 44 countries, 36% white, 43% black, 10% Hispanic and 11% other). 47% were obese (BMI > 30 kg/m2). 72% of tumours were ER+ and/or PR+, 20% were Tneg and 13% were HER2+. The odds of having a Tneg tumour were 3-fold higher (95% CI 1.6, 5.5; p = 0.0001) in black compared with white women. Tneg tumours were equally common in black women diagnosed before and after age 50 (31% vs 29%; p = NS), and who were obese and non-obese (29% vs 31%; p = NS). Considering all patients, as BMI increased, the proportion of Tneg tumours decreased (p = 0.08).

**Conclusions:**

Black women of diverse background have 3-fold more Tneg tumours than non-black women, regardless of age and BMI. Other factors must determine tumour subtype. The higher prevalence of Tneg tumours in black women in all age and weight categories likely contributes to black women's unfavorable breast cancer prognosis.

## Introduction

Breast cancer is a clinically and genetically heterogeneous disease, varying substantially in incidence and mortality according to race/ethnicity [1]. To better understand the clinical and pathological features associated with breast cancer, we created a database of all invasive breast cancer patients seen at our institution. The Boston University Medical Center includes a tertiary-care hospital that is the largest safety net provider in New England and provides care to a diverse population. Approximately 75% of patients are insured under government-funded programs (e.g., Medicaid or Medicare) or receive free care. One-half have an annual income below $20,420 and 30% do not speak English (medical interpreters deliver translation in 60 languages). The institution's patients includes self-identified ethnic groups in the following proportions: 36% black, 32% white, 16% hispanic, 4% Asian and 13% other. "Asian" here includes most South-East Asian nationalities, except Thailand, Singapore, Taiwan. Those nationalities, along with Indian, are included in "other".

In the present study, we used the database to examine the incidence of triple-negative breast cancers and their associated clinical and pathological features.

The triple-negative immunophenotype, that is, oestrogen receptor (ER), progesterone receptor (PR) and human epidermal growth factor receptor (HER) 2 negative, constitutes approximately 15% of all invasive breast cancers. It is often categorised as a basal-like tumour, a distinct biological subtype identified by gene expression. Many basal-like tumours express cytokeratins (CK) 5, 6 and 17 as well as HER1 (epidermal growth factor receptor (EGFR) [2]. Basal-like tumours are associated with aggressive histological features [3], BRCA mutation carriers [4] and poor prognosis [4,5]. Basal-like tumours are more common in premenopausal African-American women compared with postmenopausal African-American women or non-African-American women [6]. These reports led us to investigate the proportions of triple-negative tumours in our ethnically heterogeneous population and to evaluate whether these triple-negative tumours also belonged to the basal-like subtype.

In addition, we queried whether body mass index (BMI) was associated with triple-negative tumours. Elevated BMI has been shown to be associated with an increased risk of hormone receptor-positive breast cancer in postmenopausal women [7,8]. In general, obesity has been shown to be associated with an increased risk of breast cancer and decreased survival [9], with a worse prognosis in both pre- and postmenopausal women [10,11]. Both white [12-14] and black premenopausal women [15] have modest inverse associations between body weight and breast cancer incidence [16,17]. Postmenopausal obese women have an increased risk of developing breast cancer and decreased survival [18,19].

The overall incidence of breast cancer among black women in the USA is lower than in white women [20]. However, black women are more likely to have advanced stage of disease at diagnosis, higher risk of recurrence and worse overall prognosis [21-23]. The reasons for this difference are likely to be multifactorial. Some studies suggest that one factor may be a variation in obesity and body fat distribution between black and white women [24-26]. There are few reports on the associations between race/ethnicity, BMI, age and breast cancer subtypes. Reports on the relation between BMI and triple-negative breast cancer specifically are even fewer. To elucidate potential relations, we investigated associations between clinical features (race/ethnicity, BMI, age) and tumour characteristics (grade, ER, PR and HER2 status, nodal involvement).

## Materials and methods

### Study population

With Institutional Review Board approval, we established a database of all female patients diagnosed at our institution with invasive breast cancer between March 1998 and November 2006. Informed consent was waived because individual patient information was identified only by investigator-generated code numbers that are not linkable to patient identifiers. For each patient, we identified tumour grade, stage, level of ER, PR and HER2 expression (or gene amplification), patient age, BMI (using standard National Heart, Lung and Blood Institute categories) and self-identified racial group. Recurrent tumours were excluded. (Recurrence was defined by time elapsed since first tumour, interim treatment, immunophenotype, histology, metastases and clinical impression). Ten of 415 tumours (2%) were second primary or synchronous contralateral breast tumours and were included in this study.

### Data collection

We queried electronic medical records (EMR) and performed manual medical record review for all patients to ensure quality control. Tumour histology, grade, stage, ER, PR and HER2 expression were determined from the original pathology reports. Tumours had been diagnosed by experienced pathologists using standard criteria for histology and modified Scarff-Bloom-Richardson criteria for grade. ER and PR expression were determined using immunoperoxidase staining (Dako, Carpinteria, CA, USA) and quantified by image analysis (Biogenex, San Ramon, CA, USA) with values less than 5% categorised as negative. HER2 expression was determined by immunohistochemistry (Dako, Carpinteria, CA, USA). Tumours that showed 2+ Her2/neu immunohistochemistry staining based on HercepTest criteria were definitively assessed by fluorescence *in situ *hybridisation (FISH) (Vysis, Des Plaines, IL, USA). Tumours were designated as being HER2+ if they showed 3+ Her2/neu immunohistochemistry staining (based on HercepTest criteria) or if they were FISH positive [27,28]. Stage at diagnosis was coded according to the *American Joint Commission on Cancer's Cancer Staging Manual *[29].

Patient age at diagnosis was calculated using the dates of birth and diagnosis in the EMR. Patients were categorised as diagnosed at age 50 years or younger, when they are more likely to be premenopausal, or at age over 50 years, when they are more likely to be postmenopausal. BMI was calculated by weight (kg)/height squared (m^2^) using EMR data. In 97% of cases, weight and height were recorded within six months of diagnosis. If more than one weight or height value was available, we used the values closest to the date of diagnosis. Patients were placed into one of five BMI categories: under/normal weight (BMI <25), overweight (BMI 25 to <30), obesity I (BMI 30 to <35), obesity II (BMI 35 to <40) or obesity III (BMI ≥ 40).

Racial/ethnic group was determined by patient self-identification at the time of registration. Categories included: white/caucasian, black/African-American, hispanic, Asian/Pacific Islander, Middle Eastern and other. Due to sparse data, patients self-identified as Asian or Middle Eastern were included in the other category. To test the accuracy of the registration data, a manual review of the EMR was performed to determine provider-identified racial/ethnic group. A provider-identified racial/ethnic group was available for 402 of 415 patients and was concordant with the self-identified group in 91% of cases. When groups differed, the self-identified group was used. EMR data often included country of origin.

Because women born in the Caribbean constituted a large subgroup of our black population (n = 56, 27%), we compared this subgroup to the rest of the black population, which comprised patients who were provider-identified as African-American, African or black without further specification. Our Caribbean black patients were from countries with a majority pan-West African origin (Haiti, Jamaica, Trinidad, Tobago, Barbados and Montserrat). Patients from the Caribbean countries with a majority hispanic origin (Dominican Republic, Puerto Rico and Cuba) were classified as hispanic and not included in this black subgroup comparison.

### Morphology and immunohistochemistry

For 56 of the 81 triple-negative tumours, residual paraffin-embedded tumour tissue was available and additional evaluation could be performed. For additional morphological assessment, tumours were classified as grade 2 or grade 3 ductal carcinoma not otherwise specified (NOS), medullary-like carcinoma or other (for example, grade 1 ductal NOS, lobular or micropapillary tumours). Additional immunohistochemistry was performed with antibodies to CK 5/6 (Biocare Medical, Concord, CA, USA), and EGFR (Dako, Carpinteria, CA, USA) using a streptavidin-biotin horseradish peroxidase detection kit (Biogenex, San Ramon, CA, USA). The expression of these markers was graded semi-quantitatively. Allred scoring [30] was used for CK5/6, with a score of 5 or more considered as positive. HercepTest scoring criteria [27] were used to grade EGFR expression, with a score of 2+ or more considered as positive [31].

### Statistical analysis

Data on patient and tumour characteristics were entered into a Microsoft Excel worksheet (Redmond, WA, USA) and exported into the SAS (Cary, NC, USA) statistical package. We used chi-squares statistics based on contingency tables to test for homogeneity of proportions and multivariate logistic regression to determine associations between patient-tumour characteristics and triple-negative breast cancer. Odds ratios (OR) and 95% confidence intervals (CI) from the multivariate logistic regression analyses were adjusted for race, BMI and age (≤ 50 or > 50). We investigated whether there was an adjusted association between categorical variables (race and BMI group) and triple-negative tumour type by calculating twice the difference in the model log-likelihood with and without the categorical variable. This was distributed as chi-squared with degrees of freedom equal to the number of categories less one. Women with missing data were excluded from these analyses.

## Results

### Patient characteristics

**Figure 1:**
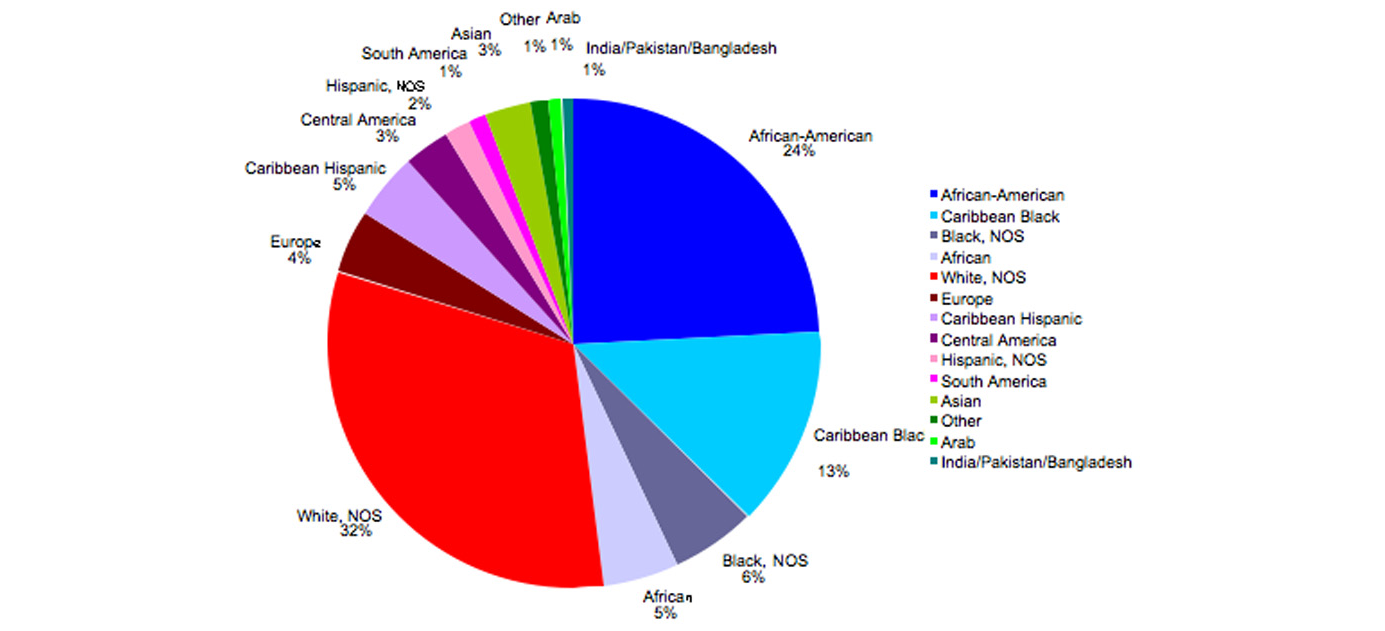
**Distribution of breast cancer patients by race/ethnicity**. The chart depicts the proportion of patients by race/ethnicity, classifying them by region of origin. NOS, not otherwise specified.

The demographic features of the 415 patients in our database are presented in Table 1. Some features are consistent with averages for the USA, but our population is unusual compared with most other reported cohorts because of its marked ethnic and racial diversity (Figure 1). In the present analysis, we grouped patients into four racial/ethnic categories: white (36%), black (43%), hispanic (10%) and other (11%). Within these broad categories, our patient population contained diverse subcategories. The median age at diagnosis of invasive breast cancer in our patients was 58 years, which is slightly younger than the US average age of 61 years, as reported in the National Cancer Institute's SEER Cancer Statistics Review [32]. Of our patients, 29% were 50 years or younger and 71% were older than 50 years at diagnosis. These proportions are generally consistent with US averages [33]. Notable in our population was the frequency of elevated BMI (only 23% of women fell into the under/normal weight category, while 30% were overweight, 27% were classified as obesity I, 13% as obesity II and 7% as obesity III).

**Table 1:** Association between race/ethnicity and clinical-pathological features

Category (p-value)*	**White**	**Black**	**Hispanic**	**Other**	**All**
	N (%)				

**Race**	148 (36)	177 (43)	43 (10)	47 (11)	415

**Age (years) (0.04)**					
**≤50 (premenopausal)**	37 (25)	53 (30)	20 (47)	15 (32)	125 (30)
**>50 (postmenopausal)**	111 (75)	124 (70)	23 (53)	32 (68)	290 (70)

**ER positive (0.0003)**	115 (80)	104 (59)	35 (81)	35 (74)	289 (70)

**PR positive (<0.0001)**	105 (72)	90 (51)	36 (84)	22 (47)	253 (62)

**Her2 positive (0.03)**	11 (7.5)	26 (15)	5 (12)	11 (23)	53 (13)

**Triple negative (0.0002)**	19 (13)	52 (30)	5 (12)	5 (11)	81 (20)

**Grade (0.01)**					
1	27 (19)	14 (8.5)	7 (16)	6 (13)	54 (14)
2	70 (49)	72 (44)	20 (46)	14 (31)	176 (45)
3	45 (32)	78 (48)	16 (37)	25 (56)	164 (42)

**T (tumour size) (0.64)**					
1	76 (58)	94 (58)	22 (54)	22 (52)	214 (57)
2	44 (33)	56 (35)	12 (29)	15 (36)	127 (34)
3	9 (6.8)	12 (7.4)	6 (15)	4 (9.5)	31 (8)
4	3 (2.3)	0 (0)	1 (2.4)	1 (2.4)	5 (1)

**No. of positive nodes (0.50)**					
0	64 (59)	66 (47)	18 (56)	22 (56)	170 (53)
1 to 3	27 (25)	43 (31)	11 (34)	8 (21)	89 (28)
4 to 9	11 (10)	20 (14)	2 (6)	8 (21)	41 (13)
≥ 10	6 (6)	11 (8)	1 (3)	1 (3)	19 (6)

**BMI (kg/m**^2^**) (0.01)**					
Underweight/normal	39 (27)	32 (18)	5 (12)	19 (40)	95 (23)
Overweight	45 (31)	47 (27)	19 (44)	11 (23)	122 (30)
Obesity I	32 (22)	55 (31)	11 (26)	13 (27)	111 (27)
Obesity II	20 (14)	26 (15)	3 (7)	2 (4.3)	51 (13)
Obesity III	7 (4.9)	15 (8.6)	5 (12)	2 (4.3)	29 (7)

* test of homogeneity of proportions

BMI = body mass index; ER = oestrogen receptor; HER = human epidermal growth factor receptor; PR = progesterone receptor.

### Tumour characteristics

Tumour characteristics are presented in Table 1. Of the 415 invasive breast cancers in our database, 81 (20%) were triple negative, 297 (72%) were ER and/or PR positive and 53 (13%) were HER2 positive. Overall, our patients had fewer HER2-positive tumours and more triple-negative tumours than in some series, although our proportions are consistent with those in racially/ethnically heterogeneous cohorts [34-36]. In addition, our patients presented at a somewhat later stage than average for the USA: 47% of our tumours were lymph node-positive; the USA average is 39% [32]. However, the presence of 96 tumours with unconfirmed node status may have affected these proportions.

We characterised all available triple-negative tumours (56 of 81 (69%)) with additional morphological classification and immunohistochemical staining for CK5/6 and EGFR to determine what proportion of the triple-negative tumours had the basal-like phenotype. We found that 19 of 56 (34%) tumours were medullary-like, 14 of 56 (25%) were grade 2 and 18 of 56 (32%) were grade 3 ductal carcinoma NOS and 5 of 56 (9%) were of other histologies. Of 56 tumours, 38 (68%) were CK5/6 and/or EGFR positive. There was a significant association between CK5/6 expression and EGFR expression with a Spearman correlation coefficient of 0.34 (p = 0.01). Taken together, these studies suggest that our triple-negative tumours include a high proportion of basal-like tumours.

### Patient-tumour associations

We examined associations between race, BMI, age (and presumed menopausal status), tumour grade, ER expression, PR expression, HER2 expression and nodal involvement. We confirmed previously noted associations between patient and tumour variables, for example, positive associations between markers of poor prognosis (e.g., grade and stage). Of particular relevance, we found in bivariate analyses that obesity was associated with race (p = 0.01) [15,37] and that triple-negative status was associated with race (p = 0.0002) [6,34-36,38,39].

Table 1 shows the associations of race/ethnicity with tumour prognostic markers and other clinicopathological features. We noted two associations pertinent to triple-negative tumours and BMI. First, 30% of tumours in black women were triple negative, compared with 11 to 13% of tumours in other women. Second, 55% of black women were obese, compared with 36 to 45% of other women. There was no substantial dependence on race/ethnicity categories other than black (Table 1): the results of our analyses did not substantially depend on more finely-divided race categories, as determined by visual examination of the associations and their intervals with the reference category limited to whites versus the reference category defined as non-blacks. Therefore, we combined white, hispanic and other race/ethnicity categories into a single category 'non-black', which serves as our reference group, to address two questions.

Black women were both more likely than other women to be obese and to have triple-negative tumours, so we asked whether obese black women had a higher proportion of triple-negative tumours than other obese women. As shown in Table 2, stratifying the dataset to black vs. non-black women, we found that 29% of obese black women had triple-negative tumours compared with 8.6% of obese non-black women (OR = 4.3: 95 CI = 1.8 to 10; p = 0.0004). (Using whites as the reference category, the OR = 4.2 and 95% CI = 1.6 to 13). Similarly, 31% of non-obese black women had triple-negative tumours compared with 15% of non-obese non-black women (OR = 2.7, 95% CI = 1.4 to 5.3; p = 0.003). (Using whites as the reference category, the OR = 2.5 and 95% CI = 1.2 to 5.4). These two ORs were not significantly different from one another (p = 0.41), suggesting that among black women, BMI does not appear to be associated with triple-negative status.

**Table 2:** Association between triple-negative breast cancer and race/ethnicity within strata of obese and non-obese women.

	**Black**		**Non-black**	
	n/total (%)		n/total (%)	
	BMI ≥ 30	BMI < 30	BMI ≥ 30	BMI < 30
**Tumour immunophenotype**				
Triple negative	27/94 (29)	25/79 (32)	8/93 (8.6)	20/137 (15)
Other	67/94 (71)	54/79 (68)	85/93 (91)	117/137 (85)
**Adjusted odds ratio of triple-negative tumour** **(confidence interval)**	4.3 (1.8 to 10)	2.7 (1.4 to 5.3)	1.0	1.0

BMI = body mass index.

Next, we examined associations of age at diagnosis with tumour characteristics within black and non-black women (Table 3). In contrast to previous reports [6,40], we did not find a strong association between triple-negative tumour status and younger age when we considered all patients (24% triple-negative tumours in women aged ≤ 50 years compared with 18% triple-negative women aged > 50 years; p = 0.22). Adjustment for more finely divided age categories made no difference in the estimates of association. We next considered black vs. non-black women. We found that the proportions of triple-negative tumours were similar in younger and older black women: 31% of black women aged 50 years or younger and 29% of black women aged over 50 years had triple-negative tumours (p = 0.76). In contrast, we found a marginal association between triple-negative tumours and age in non-black women: 17% of non-black women aged 50 years or younger had triple-negative tumours, compared with 10% of non-black women aged over 50 years (p = 0.11).

**Table 3:** Associations between patient and tumour characteristics in black vs non-black women diagnosed at age ≤ 50 vs > 50 years

	**Black**		**Non-black**	
Patient/tumour characteristic (p-value*)	age ≤50 years (premenopausal)	age >50 years (postmenopausal)	age ≤50 years (premenopausal)	age >50 years (postmenopausal)

	n (%)	n (%)	n (%)	n (%)

**BMI (0.03)**				
Underweight/normal (<25)	10 (20)	22 (17)	23 (33)	40 (24)
Overweight (25 to <30)	16 (31)	31 (25)	20 (29)	55 (32)
Obesity I (30 to <35)	13 (25)	42 (33)	14 (20)	42 (25)
Obesity II (35 to <40)	4 (7.8)	22 (17)	9 (13)	16 (9.4)
Obesity III (≥ 40) Missing	8 (16) 0 (0)	7 (5.6) 2 (1.6)	2 (2.9) 1 (1.5)	12 (7.1) 5 (2.9)

**Immunophenotype (<0.0001)**				
Triple negative	16 (31)	36 (29)	12 (17)	17 (10)
Other Missing	35 (69) 0 (0)	8 (70) 2 (1.6)	56 (81) 1 (1.5)	151 (89) 2 (1.2)

**Grade (0.06)**				
1	3 (5.9)	11 (8.7)	13 (19)	27 (16)
2	19 (37)	53 (42)	25 (36)	80 (47)
3 Missing	26 (51) 3 (5.9)	52 (41) 10 (7.9)	30 (44) 1 (1.5)	56 (33) 7 (4.1)

**Nodal involvement (0.03)**				
Node negative	13 (25)	48 (38)	31 (45)	51 (30)
Node positive Missing	29 (57) 9 (18)	52 (41) 26 (21)	30 (43) 8 (12)	71 (42) 48 (28)

**Total number (%) of all patients**	51 (12)	126 (30)	69 (17)	170 (41)

* test of homogeneity of proportions.

BMI = body mass index.

To further characterise the possible relations between race/ethnicity, BMI and triple-negative breast cancer, we performed multiple logistic regression analyses. Table 4 shows the adjusted OR and 95% CI from the multiple logistic regression analyses for patient characteristics of triple-negative breast cancers compared with other types of breast cancer. The odds of having a triple-negative tumour were three-fold higher (95% CI = 1.6 to 5.4) in black women as compared with white women. Mutually adjusting for race/ethnicity, BMI and age (age ≤ 50 years vs. > 50 years, as a surrogate for menopausal status), there remained a strong association between race/ethnicity and triple-negative tumours (p = 0.0001). After adjusting for race/ethnicity and age, we noted decreasing proportions of triple-negative tumours with increasing category of BMI (p = 0.08).

**Table 4:** Adjusted odds ratios for patient characteristics and triple negative immunophenotype

**Characteristics predicting** **triple negative phenotype**	**n (%)**	**Adjusted** **odds ratio§**	**95% CI**	p-value*
**Race**				0.0001
Black	177 (43)	3.00	1.6 to 5.4	
White	148 (36)	1.00	reference	
Hispanic	43 (10)	0.83	0.28 to 2.4	
Other	47 (11)	0.79	0.27 to 2.3	

**BMI (kg/m**^2^**)**				0.08
Underweight/normal (<25)	94 (23)	1.00	reference	
Overweight (25 to <30)	123 (30)	1.00	0.51 to 2.1	
Obesity I (30 to <35)	111 (27)	0.69	0.33 to 1.5	
Obesity II (35 to <40)	51 (13)	0.85	0.35 to 2.07	
Obesity III (≥ 40)	29 (7)	0.86	0.29 to 2.6	

**Age (years)**				0.22
≤ 50 (premenopausal)	120 (29)	1.40	0.81 to 2.4	
> 50 (postmenopausal)	295 (71)	1.00	reference	

§adjusted for age, race/ethnicity and BMI; * for test of model with and without the characteristic

BMI = body mass index; CI = confidence interval.

Because women of Caribbean origin constituted a large proportion of our black population (n = 56, 27%), we compared this subgroup to the rest of the black population. Controlling for age (≤ 50 years vs > 50 years), no significant differences were seen between Caribbean black women and other black women in the proportion of triple-negative tumours, BMI, grade or node involvement (see Additional Data File 1).

## Discussion

We investigated clinicopathological features of breast cancers in a patient population unusual for its racial/ethnic and socioeconomic diversity. We focused on triple-negative tumours. Our results confirmed previously described associations between patient and tumour characteristics, and uncovered new associations. In particular, we found a three-fold increased prevalence of triple-negative tumours in black women, who comprised 43% of our patients compared with non-black women. Triple-negative tumours comprised equal fractions – approximately 30% – of breast cancers in younger and older black women, regardless of their likely menopausal status. Triple-negative tumours comprised equal fractions – about 30% – of breast cancers in obese and in non-obese black women. Considering all women together, regardless of race/ethnicity and age, we did note a trend toward an inverse association between triple-negative tumours and elevated BMI. Overall, these results suggest that black women of diverse backgrounds are much more likely to be diagnosed with triple-negative tumours, and therefore a poorer prognosis, regardless of older age or higher BMI, factors that in other populations may be associated with hormone receptor-positive tumours with a better prognosis.

Our observation that both younger and older black women have increased, equivalent proportions of triple-negative tumours contrasts with results from the Carolina Breast Cancer Study. That study found a higher prevalence of basal-like tumours only in premenopausal African-American patients [6]. The contrast may be due to the unusual heterogeneity of our population, with consequent diversity of socioeconomic, lifestyle and genetic factors. Our study highlights the complexity surrounding the issue of race/ethnicity in medical research, and the potential differences in how each can be defined, measured and interpreted [41,42]. The contrast could also reflect differences between the tumours that were studied. We defined tumours by a triple-negative phenotype and did not routinely determine if they were basal-like. The triple-negative tumours we could examine further showed morphological and immunohistochemical characteristics (i.e., medullary features and increased CK5/6 and EGFR staining) in proportions similar to what has been reported elsewhere, and are consistent with estimates that 80 to 90% of triple-negative tumours are basal-like [43-45].

Perhaps our most intriguing result is the relation between BMI and tumour subtype. Existing studies reveal a complicated relation between BMI and breast cancer. Some studies find associations between increased BMI and increased risk of developing breast cancer, higher stage at diagnosis, greater likelihood of expressing markers of high cell proliferation, poorer response to neodjuvant chemotherapy and increased disease-specific mortality [46-51]. On the other hand, there is a clear association between elevated BMI and postmenopausal, ER-positive and PR-positive breast cancers [52], perhaps due to oestrogen production from adipose tissue. If obesity is important in determining hormone receptor status, then obese black and non-black women should have similar proportions of hormone receptor-negative tumours. However, we find that obese black women have four-fold more triple-negative tumours than obese non-black women. Therefore, factors other than whole body obesity must be crucial in determining hormone receptor expression. A specific type of obesity, an elevated waist:hip ratio, was associated with the basal subtype in the Carolina dataset [40], but overall, the factors that determine subtype – genetics, microenvironment, environmental or developmental exposures – remain unclear.

The lack of association between obesity and hormone receptor expression in black women may contribute to our observation that, when we considered all 415 cases, we found a trend, rather than a significant association, between increased BMI category and decreased proportions of triple-negative tumours. The inclusion of a large subset of black women (43%), for whom increased BMI is not clearly associated with hormone receptor positivity, in the dataset may prevent us from seeing the association reported in other populations between increased BMI and hormone receptor positivity.

Our observations are consistent with the lack of association seen between BMI and overall risk of postmenopausal breast cancer in the Black Women's Health Study [15]. Those authors speculated that if obesity confers mainly a risk of hormone receptor-positive tumours, then it would be difficult to detect an association between elevated BMI and postmenopausal breast cancer if a large proportion of the black women's cancers were hormone receptor negative. This is precisely our finding. Our observations are also consistent with observations that high BMI is associated with hormone receptor-negative tumours in cohorts of different ethnic composition [50,53].

Potential limitations of our study include its relatively small size and lack of data on clinical outcome or on potential confounders, such as parity. These limitations, however, are balanced by strengths: the unusual, highly heterogeneous population, data extraction from computerised records augmented with manual abstraction for quality control, and a consistent, single-institution approach to pathological diagnosis and patient care. Although we do not have the socioeconomic status of each patient, the population in the database is highly likely to reflect the institution's overall socioeconomic status data. These strengths contributed to our database confirming many previously noted associations between clinical and pathological features.

The observations we report from this database have potential clinical implications. Several studies have documented the poor outcome of patients with triple-negative or basal-like tumours [35,38], and the greater mortality of black women with breast cancer compared with other women, regardless of age [6,35,38]. Our findings that a diverse group of both younger and older, normal-weight and obese, black women have approximately three-fold more triple-negative tumours than other groups, may be one factor contributing to the unfavourable prognosis of black women with breast cancer.

## Conclusion

Black women of diverse background have three-fold more triple-negative tumours than non-black women, regardless of age and BMI. Other factors must determine tumour subtype. The higher prevalence of triple-negative tumours in black women in all age and weight categories is likely to contribute to a black women's unfavourable breast cancer prognosis.

## Abbreviations

BMI: body mass index; CI: confidence intervals; EGFR: epidermal growth factor receptor; EMR: electronic medical record; ER: oestrogen receptor; FISH: fluorescence *in situ *hybridisation; HER: human epidermal growth factor receptor; NOS: not otherwise specified; NS: not significant; OR: odds ratios; PR: progesterone receptor.

## Competing interests

The authors declare that they have no competing interests.

## Authors' contributions

LS contributed to the study's conception and design, data collection and analysis, and manuscript writing. TL contributed to the study's data collection and analysis and manuscript writing. JW and DC contributed to the study's conception and design, and to data collection and analysis. JS, RB and MC contributed to the study's data collection and assembly. JCL contributed to the study's design, data collection and analysis and contributed to the manuscript writing. TK contributed to the study's conception and design, data collection and analysis, and manuscript writing. CLR contributed to the study's conception and design, provided financial support, data analysis and interpretation and manuscript writing.

## Supplementary Material

Additional file 1 A word file containing a table that lists the associations between patient and tumour characteristics in Caribbean black women vs other black women. Click here for file
