# Supplementary material for: Triple-negative breast cancers are increased in black women regardless of age or body mass index
Source: Breast Cancer Res. 2009 Mar 25;11(2):R18. doi: 10.1186/bcr2242 (PMC2688946; doi:10.1186/bcr2242)
Supplement: Additional file 1 — A word file containing a table that lists the associations between patient and tumour characteristics in Caribbean black women vs other black women. [file bcr2242-S1.doc]

**Additonal File 1**

**Associations between patient and tumour characteristics in Caribbean black women vs. other black women (combined)**

|  | **Caribbean black** | | **Other black** | |
| --- | --- | --- | --- | --- |
| **Characteristic** | Premenopausal (age <50 years) | Postmenopausal (age >50 years) | Premenopausal (age <50 years) | Postmenopausal (age >50 years) |
|  | n (%) | n (%) | n (%) | n (%) |
| Body mass index |  |  |  |  |
| Underweight/normal (0 to <25) | 5 (24) | 7 (21) | 7 (19) | 21 (19) |
| Overweight (25 to <30) | 8 (38) | 11 (32) | 11 (30) | 25 (23) |
| Obesity I (30 to <35) | 7 (33) | 12 (35) | 8 (22) | 36 (33) |
| Obesity II (35 to <40) | 1 (5) | 4 (12) | 4 (11) | 19 (17) |
| Obesity III (≥40) | 0 (0) | 0 (0) | 7 (19) | 8 (7) |
| Immunophenotype |  |  |  |  |
| Triple negative | 8 (38) | 10 (29) | 10 (27) | 29 (27) |
| Other | 13 (62) | 25 (71) | 27 (73) | 79 (73) |
| Grade |  |  |  |  |
| 1 | 2 (11) | 2 (6) | 2 (6) | 11 (11) |
| 2 | 7 (37) | 11 (34) | 11 (31) | 48 (47) |
| 3 | 10 (53) | 19 (59) | 23 (64) | 43 (42) |
| Stage |  |  |  |  |
| Node negative | 11 (61) | 14 (48) | 21 (70) | 44 (51) |
| Node positive | 7 (39) | 15 (52) | 9 (30) | 42 (49) |
| Total number (%) of black patients | 20 (9) | 36 (18) | 36 (18) | 111 (55) |
